# Cocaine, cardiomyopathy, and heart failure: a systematic review and meta-analysis

**DOI:** 10.1038/s41598-020-76273-1

**Published:** 2020-11-13

**Authors:** Daniel J. Arenas, Sourik Beltran, Sara Zhou, Lee R. Goldberg

**Affiliations:** 1 Perelman School of Medicine, University of Pennsylvania, Philadelphia, USA; 2 Department of Medical Ethics and Health Policy, University of Pennsylvania, Philadelphia, USA; 3 College of Arts and Sciences, University of Pennsylvania, Philadelphia, USA; 4 Penn Medicine Heart Failure and Cardiac Transplant Center, Perelman Center for Advanced Medicine, 11-171 South Tower, 3400 Civic Center Boulevard, Philadelphia, PA 19104 USA

**Keywords:** Cardiology, Diseases

## Abstract

Although the cardiotoxic effects of cocaine are universally recognized, the association between cocaine and cardiomyopathy and/or heart failure is poorly understood. To conduct a comprehensive review and meta-analysis on the association between cocaine, heart failure, and cardiomyopathy, we first conducted a broad-term search in PubMed, Embase, Web of Science, and Scopus for human studies containing primary data on the relationship between cocaine and heart failure or cardiomyopathy. We were interested in studies with data beyond acute coronary syndromes. Retrieved studies were grouped into different categories based on possible hypotheses to test by meta-analysis. A second search with specific terms was then conducted. For grouped studies with sufficient clinical and methodological homogeneity, effect sizes were calculated and combined for meta-analysis by the Random Effects model. There is in general a need for more primary data studies that investigate heart failure and/or cardiomyopathy in cocaine users for mechanisms independent of ischemia. There were, however, enough studies to combine by meta-analyses that showed that chronic cocaine use is associated with anatomical and functional changes more consistent with diastolic heart failure instead of the commonly taught dilated cardiomyopathy pathway. In patients without a history of ACS, chronic cocaine use was not associated with significantly reduced EF. The few studies on acute cocaine had conflicting results on whether single-dose intravascular cocaine results in acute heart failure. Studies identified that included beta-blockade therapy in cocaine users with cardiac disease suggest that beta-blockers are not unsafe and that may be effective in the treatment of cocaine-associated heart failure. Chronic cocaine use is associated with anatomical and physiological changes of the heart muscle that are potentially reversible with beta-blockade therapy.

## Introduction

Cocaine is one of the most widely consumed recreational drugs in the world with the number of global users estimated at around 18 million^1^. In the United States, cocaine use is regarded as a major source of morbidity and mortality with effects ranging from long-term cognitive impairment to early death^2–5^. The cardiovascular effects of cocaine are of particular interest to researchers and clinicians as they account for more than 64,000 presentations of chest pain every year and an estimated $155—$226 million in annual healthcare costs^6,7^. Furthermore, cardiac complications remain some of the most frequently attributed causes of cocaine-related death^8^.

While less frequently investigated than cocaine-associated myocardial infarction^9,10^, the link between cocaine use and heart failure is commonly recognized. However, the precise clinical, physiological, and anatomical characteristics of heart failure due to cocaine remain poorly understood. In fact, despite numerous reviews exploring the relationship between cocaine and acute coronary syndrome^11–13^, there has been no systematic review or meta-analysis to date that has integrated existing data linking cocaine to cardiomyopathy and/or heart failure^14,15^.

In this systematic review and meta-analysis, we broadly evaluate the available evidence on the association between cocaine and cardiomyopathies or heart failure. We focused on studies that explored these aspects and were not simply limited to cocaine and acute coronary syndromes. Our broad search of the literature found a very limited number of studies, beyond case reports/series that investigated the association between cocaine and cardiomyopathy or heart failure/dysfunction beyond ischemic cardiomyopathy. Although the number of studies was limited, there were sufficient hypotheses, testable by meta-analysis, concerning how there are anatomical and physiological changes in the heart of cocaine users compared to non-users. We identified sufficient studies to test hypotheses on systolic function being affected by acute or chronic cocaine use; on increases in heart weights and wall thicknesses associated with cocaine use; decreases in LVED; and beta blockers being unsafe for treating chronic cocaine users with heart failure or cardiomyopathy.

## Methods

Section S1 of the supplementary documents discusses the detailed methodology. Here, we highlight the most important characteristics.

### Broad search for all available literature

Our first search was aimed at broadly characterizing all studies that explored cocaine and cardiomyopathy and heart failure. We were interested in peer-reviewed human studies, of any design and publication year, that explored these concepts. The information sources were: PubMed, Scopus, Embase, and Web of Science, all searched on 06/03/2018. All the specifics of the search terms are presented in S1.2 of the supplementary documents.

### Study selection

Every retrieved abstract was inspected by three study personnel. A study was deemed relevant if it satisfied all of the following criteria: (1) The study was concerned with a relationship between cocaine and acquired cardiomyopathy of any kind and/or acquired HF of any kind; (2) It involved human subjects; (3) It involved clinical research in any study design. Studies focusing on myocardial infarction, coronary dissection, coronary vasospasms, or valvular disease were only kept if they also studied any aspect of HF or cardiomyopathy.

Non-excluded abstracts were then text evaluated to confirm eligibility and separate primary data studies from case reports and reviews without primary data. Every step of the study selection is detailed in S1.3 of the supplementary documents and summarized in Figure S2-1.

### Grouping of studies and hypotheses potentially testable by meta-analyses

Primary data studies identified by the first search were placed into multiple overlapping groups based on data availability for measured outcomes. Section S1.4 and Tables S3.1–10 provide all data on how the studies were grouped. The groupings suggested that the following hypotheses could be tested by meta-analysis of the available literature: Systolic function is lower in chronic cocaine users; the prevalence of low LVEF in cocaine users is higher than that in non-users; acute usage of cocaine results in a measurable and significantly lower LVEF; the heart weight and wall thicknesses of cocaine users, whether measured by autopsy or by echocardiogram, is higher than those of controls; LVED is lower in users compared to in non-users; beta-blockers are not safe for use in chronic cocaine users.

To more clearly integrate the identified studies’ diverse findings, we have used the following specific language: Heart failure refers to symptoms; dysfunction refers to anatomical or measurable changes (i.e. Left ventricular ejection fraction).

### Second literature search

On 12/21/2018, we performed a supplementary search for primary data studies this time incorporating search terms specific to the measurement outcomes and modalities identified in our initial search in order to ensure that we had retrieved all articles available for our meta-analyses (i.e. “heart weight”, “LVMI”, “echocardiogram”, “cardiac magnetic resonance”). S1.5 of the supplementary documents shows the supplementary search terms and S1.6 the study inclusion process.

### Data extraction and quality assessment

For each primary data study, one researcher extracted data for each outcome and measurement modality (i.e. ejection fraction measured by echocardiogram), specifics of the population, study design, sample size, the measurement modality for cocaine use (i.e. self-reported use, urine positive test), and the primary outcomes (i.e. ejection fraction).

The data items extracted from each paper included summary measures such as prevalence, odd ratios, risk ratios, and standardized mean difference (SMD). We used Hedges’ *g* for SMDs as it contains a correction for small sample sizes and allows groups with unequal variances and size. If a study did not report these effect sizes, or their confidence intervals, we attempted to extract enough primary data to calculate the effect sizes ourselves. After tables of extracted data were created, for each group of studies, one more researcher independently confirmed the accuracy of the extracted data (Tables S3-1, S3-2 …).

All studies to be included in the meta-analyses were assessed for individual study bias; the methodology and discussion are presented in the supplementary documents (S1.9).

### Data synthesis and analysis

Synthesis of summary measures was performed by meta-analysis using the random effects size model, with the restricted maximum likelihood estimator. The heterogeneity of studies was calculated using the *Q* and *I*^*2*^ statistics. Assessment of risk across publications was explored using funnel plots and two funnel plot asymmetry tests: rank correlation and regression^16,17^. Finally, subgroup analysis was performed for each meta-analysis in which sufficient studies were available to create subgroups larger than two. Further details are presented in S1.11–13 of the supplementary documents.

## Results

The broad systematic search of the literature yielded 881 abstracts, 104 of which were text evaluated. Most of these manuscripts were either case reports (41) or reviews (39), with twenty primary-data manuscripts were identified at this point. The second search, with terms specific to the hypotheses involving anatomical and functional cardiac changes, added fourteen more manuscripts. The studies were then divided into the following groups: investigation of prevalence of low LVEF in asymptomatic and symptomatic chronic cocaine users (Tables S3-1 and S3-2); acute changes in LVEF after cocaine infusion (Table S3-3); cohort studies comparing LVEF in cocaine users and non-users (Tables S3-4 and S3-5); cohort studies investigating heart weight (Table S3-6), left ventricular end diastolic volume (LVED) (Table S3-7), wall thicknesses (Table S3-8); treatment studies (Table S3-9); and other findings (Table S3-10). It should be emphasized that the identified hypotheses involved anatomical and/or measurable parameters and were therefore concerned about systolic or diastolic dysfunction. The only studies discussed in this review that addressed symptoms were the treatment studies (Table S3-9).

### Prevalence of low LVEF

Three cross-sectional studies have measured the prevalence of left ventricular dysfunction (LVEF < 50%) among asymptomatic chronic cocaine users^18–20^. (Table S3-1). The mean prevalence varied from 0 to 5%, all with overlapping confidence intervals. Meta-analysis using the random-effects model yielded an average prevalence of 0.021 [95% CI: 0.0–0.074, *n* = 219, *Q*(*df* = 2) = 7, *I*^2^ = 70%]. (Figure S2-3). Three studies, with a total of 351 patients, presented data on the prevalence of low LVEF in cocaine users who specifically presented to the emergency department with chest complaints^9,21,22^ (Table S3-2). The observed prevalence ranged from 2 to 54%. The difference in chest complaints between the three studies varied considerably and was not deemed suitable for meta-analysis.

### Cocaine and left ventricular ejection fraction

Two studies have investigated the immediate impact of cocaine use on LVEF (Table S3-3). Eisenberg et al*.* reported no significant statistical difference in LVEF after a single peripheral IV infusion of a recreational dose of cocaine in 20 volunteers with a history of cocaine use^23^. Then, Pitts et al*.* performed a similar experiment in 20 patients undergoing cardiac catheterization for the evaluation of chest pain. In this experiment there was a significant drop in LVEF following direct coronary infusion of cocaine by catheterization^24^. The difference in cocaine administration, and presentation (asymptomatic versus symptomatic), makes a comparison difficult.

**Figure 1:**
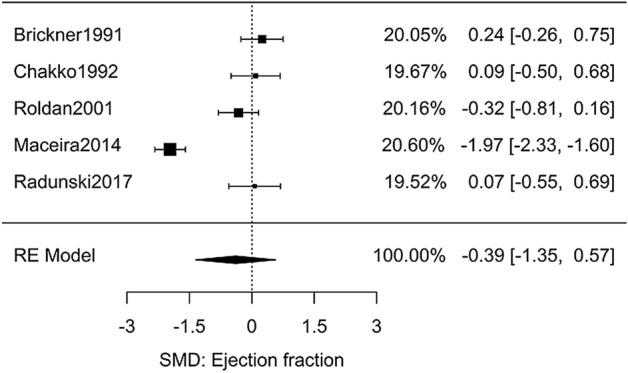
Meta-analysis of studies investigating the effect of chronic cocaine use on LV function.

Regarding chronic use, five retrospective cohort studies compared the left ventricular ejection fractions between asymptomatic adult cocaine users and non-users (Table S3-4)^25–29^. Fig. 1 shows that meta-analysis yielded a non-significant effect size *g* = − 0.39 [95% CI: − 1.35 to 0.57, *n* = 407, *Q*(*df* = 4) = 74, *I*^2^ = 95%]. The outlier study was that of Maceira^28^. Interestingly, even though all participants in this study were asymptomatic, this was the only manuscript that did not report ACS as an exclusion criterion. Removal of this study resulted in an effect size of *g* = 0.00 [95% CI: − 0.27, 0.27, *n* = 257, *Q*(*df* = 3) = 2.7, *I*^2^ = 0] which remained statistically insignificant but displayed heterogeneity less than that expected from random chance (Figure S2-4).

Regarding symptomatic users, three studies compared the ejection fraction of symptomatic chronic cocaine users versus non-users. Due to differences in severity of patient complaints, meta-analysis was deemed inappropriate (Table S3-5)^30–32^.

### Cocaine and heart weight

**Figure 2:**
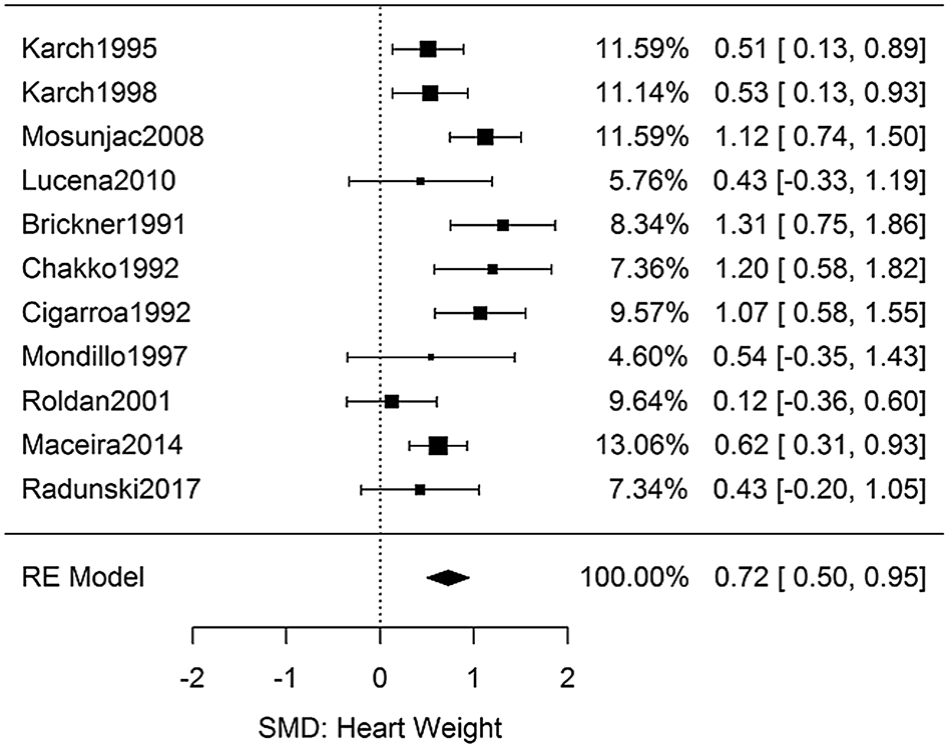
Meta-analysis of studies investigating the effect of chronic cocaine use on heart weight.

Six autopsy studies were identified which compared the heart weights of chronic cocaine users to non-users^33–38^. Table S3-6 shows the studies along with the effect sizes calculated from the primary data in each manuscript. Eight echocardiogram studies examined the left ventricular mass index (LVMi) of chronic users versus non-users^25–29,39–41^. Three studies were discarded from meta-analyses due to unmatched groups^34,38,39^. The results (Fig. 2) yielded a combined Hedges’ *g* effect size of 0.72 [95% CI: 0.50–0.95, *n* = 875, *Q*(*df* = 10) = 23, *I*^2^ = 56%]. Beyond the utility of testing for statistical significance, an SMD of 0.72 relates that an individual at the median of heart weights in the cocaine group would be in the top 25% of the comparison group.

There were sufficient studies to perform subgroup analyses by dividing the two measurement modalities: autopsy and echocardiogram (Figure S2-5). The two modalities yielded nearly identical effect sizes and heterogeneities: *g* = 0.68 [95% CI: 0.35–1.02, *Q*(*df* = 3) = 6.9, *I*^2^ = 56%] and *g* = 0.75 [95% CI: 0.43–1.08, *Q*(*df* = 6) = 16, *I*^2^ = 62%].

### Cocaine and left ventricular end diastolic volume

**Figure 3:**
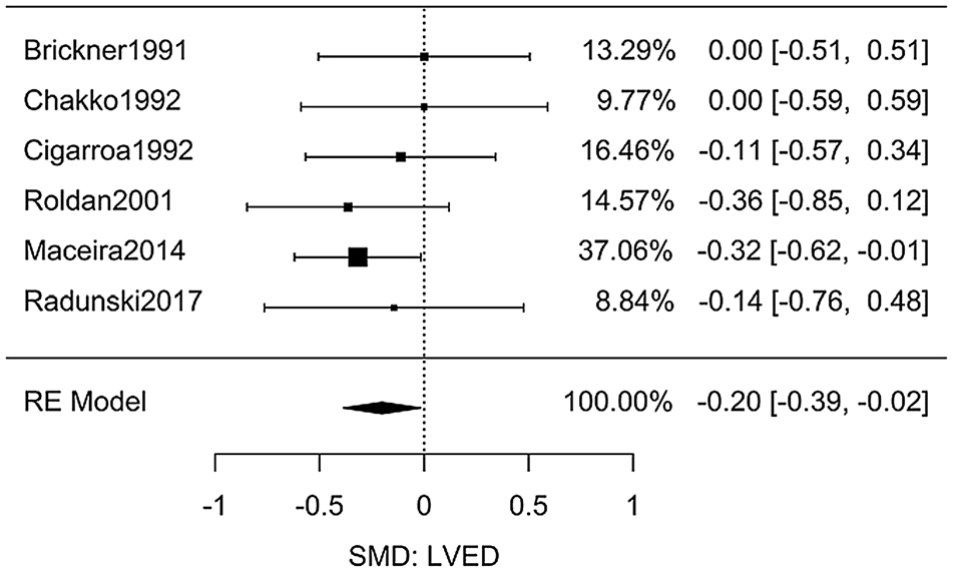
Meta-analysis of studies investigating the effect of chronic cocaine use on LVED.

Six studies (Table S3-7) had sufficient primary data to extract the effect size of chronic cocaine use on left ventricular end diastolic volume (LVED)^25–29,40^. The difference in LVED between the user and non-user groups was significant as shown in Fig. 3, *g* = − 0.20 [95% CI: − 0.39 to − 0.02, *n* = 486, *Q*(*df* = 5) = 2.2, *I*^2^ = 0%]. Although significant, the effect size was not as large as the heart weight measurements; a SMD of − 0.20 describes that the average cocaine user is in the bottom 40th percent of a comparable non-user group.

### Cocaine and relative wall thickness

**Figure 4:**
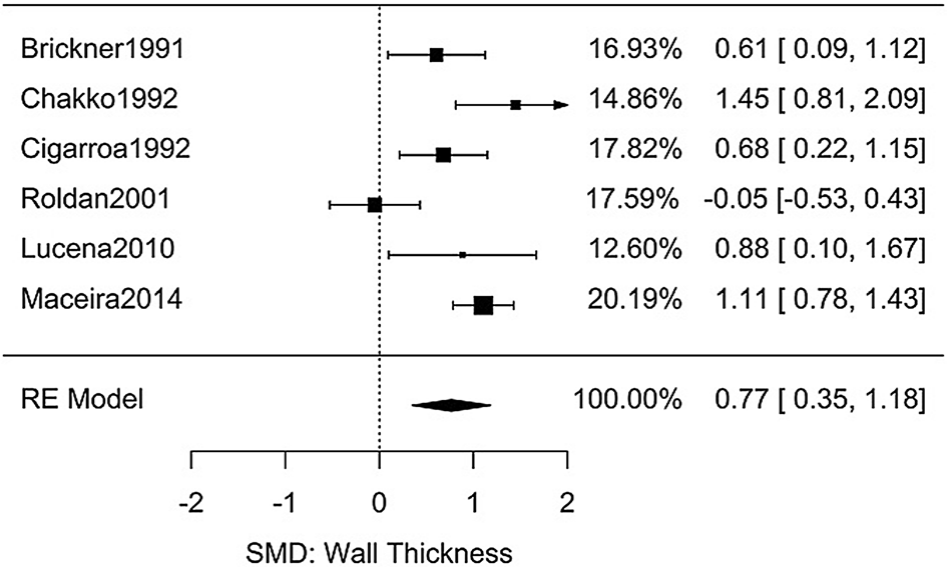
Meta-analysis of studies investigating the effect of chronic cocaine use on relative wall thickness.

For relative wall thickness (RWT), six studies contained sufficient data to extract differences in chronic cocaine users compared to non-users (Table S3-8)^25–28,37,40^. Meta-analysis (Fig. 4) showed significantly higher RWT in the user groups, *g* = 0.77 [95% CI: 0.35–1.18, *n* = 601, *Q*(*df* = 5) = 20, *I*^2^ = 75%].

Other retrieved markers of diastolic dysfunction were fractional shortening and the Doppler E/A ratio. Two studies demonstrated a significant decrease in FS although with high heterogeneity: g = − 0.65 [95% CI: − 1.05 to − 0.26, *n* = 126, *Q*(*df* = 1) = 11, *I*^2^ = 91%] (Figure S2-6). Synthesis of three studies with E/A data resulted in a non-significant change *g* = − 0.22 [95% CI: − 0.57 to 0.12, *n* = 153, *Q*(*df* = 2) = 1.5, *I*^2^ = 0%] (Figure S2-7).

### Treatment of cocaine-associated heart failure

Five articles were identified which investigated the treatment of patients with HF in the context of chronic cocaine use (Table S3-9). One article was excluded as its focus was cocaine abstinence^42^, and two had overlapping data^43,44^. All were retrospective cohort studies. Inspection of the four studies by the Newcastle–Ottawa Rating scale of non-randomized studies resulted in no manuscripts with severe concerns worthy of removal from this review.

The first study by Nguyen et al. compared the rates of adverse events between 90 active chronic cocaine users and 177 non-cocaine users while on beta-blocker therapy. They found no statistically significant difference in rates of cardiovascular adverse events, all-cause mortality, or all-combined adverse-outcomes^45^. Additionally, the authors performed sub-group analysis and separated the 90 active chronic users into those treated with either selective beta-blockers or non-selective beta-blockers; there was no significant difference in the mortality rates. This subgroup analysis was extremely important since the original hypothesis for counter-indicating beta blocker treatment in active cocaine users was unopposed alpha stimulation; that is, that blockage of β2 receptors would result in increased total peripheral resistance additive to the effects of cocaine on alpha receptors.

Two retrospective cohort studies were subsequently published. One study of 268 cocaine users with HF, and measured reduced ejection fraction, found that patients on beta-blockade therapy had a significantly reduced 30-day hospital readmission rate and significantly lower baseline rates of general cardiovascular comorbidities^46^. However, beta-blockers did not appear to reduce 1-year mortality rates. The second study by Lopez et al. found that, in a cohort of 72 cocaine-using patients with HF and reduced ejection fraction, 12 months of beta-blocker therapy led to significantly reduced New York Heart Association (NYHA) functional class as well as significantly improved ejection fraction^43,44^.

## Discussion

Our systematic review of the literature revealed a limited amount of primary data studies exploring the association between cocaine and cardiomyopathy or heart failure. Our first broad search allowed us to identify hypotheses potentially testable by pooling of multiple studies. Meta-analyses found that chronic cocaine use was associated with several anatomical and physiological changes linked to diastolic dysfunction including increased heart weight, decreased LVED, and increased ventricular wall thickness. The fact that all three physiologically-related parameters were impacted adds to the validity of the results. These findings suggest that chronic cocaine use may lead to concentric ventricular hypertrophy as well as decreased ventricular compliance leading to inadequate diastolic filling. Although our study highlights the need for more studies exploring markers of diastolic dysfunction, this systematic reviews suggests that, independent of ischemic heart disease, chronic cocaine use results in anatomical changes that act as direct pathway towards diastolic HF.

For patients presenting to emergency departments with chest complaints, the data suggests that cocaine users have EFs lower than non-users presenting with the same symptoms. While data are limited, this suggests that cocaine use may have a role in impairing systolic ventricular function among patients with symptomatic cardiac disease. A mechanism for LV dysfunction could be cocaine-induced small vessel coronary disease or direct myocardial damage^32^.

In contrast, our meta-analysis on studies about asymptomatic users showed that there was no significant difference in EF compared to non-users. Also notable, studies which investigated the immediate effects of intravascular cocaine on ventricular contractility found conflicting results on whether cocaine use is associated with the acute development of systolic dysfunction^23,24^. Despite the common teaching that cocaine is one of the causes of dilated cardiomyopathy, our review did not identify enough primary data to conclude that either single dose or chronic cocaine use impair left ventricular systolic function outside of coronary disease. This negative finding could be in part due to the fact that there were few studies that presented data for systolic function in cocaine users without previous coronary disease. Further research is needed as there are several additional proposed mechanisms on how cocaine can lead to cardiac injury such as catecholamine surge^47,48^, impaired intracellular calcium transport^49,50^, myocarditis^51^, and direct apoptotic effects^52^.

In terms of treatment, our review found insufficient primary data to support the hypothesis that beta-blockers are unsafe in the treatment of systolic HF among chronic cocaine users. While the use of beta-blockers in cocaine users has been the subject of controversy due to the theoretical risk of unopposed alpha adrenergic activity, all identified studies that investigated the treatment of systolic HF in chronic cocaine users failed to demonstrate an association between beta-blockers and increased risk of adverse outcomes among cocaine users. Instead, the available studies have shown reduced hospital readmission rates, reduced 30-day mortality, and demonstrable improvements in patient outcomes^44–46^. Nevertheless, the studies identified on beta-blocker therapy among chronic cocaine users with heart failure showed great heterogeneity in both patient population and methodology. Such heterogeneity is unsurprising considering the identified studies were some of the first to assess the utility of beta-blocker therapy among cocaine users and were therefore designed around a series of still evolving hypotheses. The availability of so few studies does however make it difficult to draw any firm conclusions regarding the specific benefits or risks of beta-blocker therapy in chronic cocaine users.

### Strengths and limitations

A strength of our systematic review was the use of broad criteria in the first search. This allowed us to identify the types of summary measures that could be combined by meta-analysis and the potential hypotheses to be tested. We then ensured a high sensitivity by performing a second search using those specific summary measures. Performing only one specific search would have narrowed the scope of our review. Conversely, conducting an initial search using all possible summary measures and measurable cardiac quantities could have resulted in far too many abstracts to reasonably explore.

Next, calculation of Hedges’ *g* in the meta-analyses demonstrated the immense utility of this metric by yielding a high level of concordance between effect sizes for heart weight (by autopsy) and LVMi by echocardiogram. The usage of this effect size may serve as a strength in future meta-analyses that are challenged by methodological heterogeneity, in this case at least for measurement modality^53^.

The major limitation is the availability of primary data in the literature. The original searches showed that the largest number of articles were case reports and reviews, none of which were subsequently included. This limitation also translates to concerns about the evaluation of publication bias: while the two funnel plot asymmetry tests (Figures S2-8 and S2-9) were negative, the statistical power of these tests in detecting publication bias is very low for a meta-analysis with a small (< 10) number of studies^54,55^. There was also insufficient published data to explore a temporal relationship between cocaine use and the cardiac effects hereby discussed.

Many of the cited studies do not involve analysis of non-participating subjects; if recent or heavy use of cocaine impacted patients' ability and/or willingness to take part in a study, biased selection of patients with milder cocaine use may underestimate the prevalence or severity of cardiac dysfunction or remodeling among cocaine users. Lastly, we did not a priori register the review in Prospero. Before our original search, we had no knowledge on which health outcomes would have enough studies in the literature with sufficient primary data for calculating effect sizes suitable for meta-analysis. Therefore, the beginning of the review had to be very broad and we did not find it suitable for a registration service that usually requires a priori knowledge of the specific health outcomes of interest.

Despite these limitations, this review is the first systematic review and meta-analysis attempting to better understand the relationship between cocaine use and HF or cardiomyopathy.

## Conclusions

There is a great need for more primary data studies investigating the association between cocaine and cardiomyopathy or heart failure. Our meta-analyses showed that chronic cocaine use is associated with anatomical and physiological changes consistent with diastolic dysfunction that may result in diastolic heart failure. Despite the common teaching that cocaine causes of dilated cardiomyopathy, our review did not identify enough primary data to conclude that either single dose or chronic cocaine use can affect left ventricular function outside of acute myocardial infarction; further studies would be useful to evaluate other potential mechanisms leading to systolic dysfunction^52^.

The implications of these findings are broad. First, primary care physicians may understand cocaine as an important cause of diastolic HF. Second, we may need to consider that previous history of ACS is important in evaluating the type of heart failure the chronic cocaine user may be experiencing or is at risk for. Third, physicians managing HF may use our findings to prioritize screening and treatment of cocaine addiction in an effort to mitigate anatomical and physiological changes on the heart. Next, on a population scale, public health workers or researchers may use our results to recognize chronic cocaine use as an empirically-viable point of intervention for reducing the global burden of diastolic HF.

Regarding beta-blockers, our results indicate a need to strongly consider departing from the long-held assumption that beta-blocker therapy is dangerous among cocaine users. The beta-blocker question must be answered with further research so that clinicians do not unnecessarily delay a potentially effective treatment of cocaine-associated HF.

In summary, the association between cocaine and HF or cardiomyopathy remains a complicated area. More research is needed to better understand the pathophysiological progression, prognosis, dose–response relationship, and its potential for reversibility.

## Supplementary information

Supplementary Information 1.

Supplementary Information 2.

Supplementary Information 3.
