# Supplementary material for: Cocaine, cardiomyopathy, and heart failure: a systematic review and meta-analysis
Source: Sci Rep. 2020 Nov 13;10:19795. doi: 10.1038/s41598-020-76273-1 (PMC7666138; doi:10.1038/s41598-020-76273-1)
Supplement: Supplementary file 1 — Supplementary Information 1. [file 41598_2020_76273_MOESM1_ESM.pdf]

Cocaine, cardiomyopathy, and heart failure: A systematic review and meta-analysis

Supplementary Document S1:

Additional Methodology Information

Daniel J. Arenas, PhD[a]
Sourik Beltran, BA[a,b]
Sara Zhou, BA[c]
Lee R. Goldberg, MD, MPH, FACC[a]

[a]Perelman School of Medicine at the University of Pennsylvania
[b]Department of Medical Ethics and Health Policy, University of Pennsylvania
[c]College of Arts and Sciences, University of Pennsylvania

Corresponding Author:
Lee R. Goldberg, MD, MPH, FACC
Penn Medicine Heart Failure and Cardiac Transplant Center
11-171 South Tower, Perelman Center for Advanced Medicine
3400 Civic Center Blvd
Philadelphia PA, 19104
Office: (215) 615-0820
Fax: (215) 615-0828
Email: Lee.Goldberg@pennmedicine.upenn.edu

OTHER AUTHOR CONTACT INFORMATION:
Daniel J. Arenas: Daniel.Arenas@pennmedicine.upenn.edu
Sourik Beltran: Sourik.Beltran@pennmedicine.upenn.edu
Sara Zhou: sarazhou@sas.upenn.edu

S1.1: Nomenclature

To more clearly integrate the identified studies' diverse findings, we have used specific language to discuss the precise conclusions of our review. Heart failure refers to symptoms; dysfunction refers to anatomical or measurable changes (i.e. Left ventricular ejection fraction).

S1.2: Broad search for cocaine and heart-failure/cardiomyopathy. 06/03/2018.

Four databases: PubMed, Embase, Web of Science, and Scopus, were searched for an extensive list of terms sensitive for cocaine and heart failure or cardiomyopathy. The multiple databases were chosen to avoid the potential of missing relevant studies when only one database is used.[1] Smaller subsets of these databases have been utilized and deemed sufficient in previous meta-analysis involving heart disease.[2–4]

We searched each database for the concepts: cocaine and heart failure (HF) or cardiomyopathy. For each concept, several search terms were used that were based on the medical subject headings (MeSH) suggested by the National Library of Medicine.

**Cocaine**		**Heart Failure or Cardiomyopathy**	
Cocaine		**Heart Failure**	**Cardiomyopathy**
Cocaine Hydrochloride		Heart Failure	Cardiomyopathy
Hydrochloride, Cocaine		Cardiac Failure	Cardiomyopathies
Cocaine HCl		Heart Decompensation	Myocardial Diseases
HCl, Cocaine		Decompensation, Heart	Disease, Myocardial
		Heart Failure, Right-Sided	Diseases, Myocardial
		Heart Failure, Right Sided	Myocardial Disease
		Right-Sided Heart Failure	Myocardiopathies
		Right Sided Heart Failure	Myocardiopathy
		Myocardial Failure	Cardiomyopathies, Secondary

		Congestive Heart Failure	Cardiomyopathy, Secondary
		Heart Failure, Congestive	Secondary Cardiomyopathies
		Heart Failure, Left-Sided	Secondary Cardiomyopathy
		Heart Failure, Left Sided	Secondary Myocardial Diseases
		Left-Sided Heart Failure	Disease, Secondary Myocardial
		Left Sided Heart Failure	Diseases, Secondary Myocardial
			Myocardial Disease, Secondary
			Secondary Myocardial Disease
			Myocardial Diseases, Secondary
			Cardiomyopathies, Primary
			Cardiomyopathy, Primary
			Primary Cardiomyopathies
			Primary Cardiomyopathy
			Primary Myocardial Diseases
			Myocardial Diseases, Primary
			Disease, Primary Myocardial
			Diseases, Primary Myocardial
			Myocardial Disease, Primary
			Primary Myocardial Disease

Search entries for PUBMED

Cocaine: (((((Cocaine) OR Cocaine Hydrochloride) OR Hydrochloride, Cocaine) OR Cocaine HCl) OR HCl, Cocaine)

Cardiomyopathy OR Heart Failure: ((((((((((((((((((((((((((((((Cardiomyopathy) OR Cardiomyopathies) OR Myocardial Diseases) OR Disease, Myocardial) OR Diseases, Myocardial) OR Myocardial Disease) OR Myocardiopathies) OR Myocardiopathy) OR Cardiomyopathies, Secondary) OR Cardiomyopathy, Secondary) OR Secondary Cardiomyopathies) OR Secondary Cardiomyopathy) OR Secondary Myocardial Diseases) OR Disease, Secondary Myocardial) OR Diseases, Secondary Myocardial) OR Myocardial Disease, Secondary) OR Secondary Myocardial Disease) OR Myocardial Diseases, Secondary) OR Cardiomyopathies, Primary) OR Cardiomyopathy, Primary) OR Primary Cardiomyopathies) OR Primary Cardiomyopathy) OR Primary Myocardial Diseases) OR Myocardial

Diseases, Primary) OR Disease, Primary Myocardial) OR Diseases, Primary Myocardial) OR Myocardial Disease, Primary) OR Primary Myocardial Disease))) OR ((((((((((((((((Heart Failure) OR Cardiac Failure) OR Heart Decompensation) OR Decompensation, Heart) OR Heart Failure, Right-Sided) OR Heart Failure, Right Sided) OR Right-Sided Heart Failure) OR Right Sided Heart Failure) OR Myocardial Failure) OR Congestive Heart Failure) OR Heart Failure, Congestive) OR Heart Failure, Left-Sided) OR Heart Failure, Left Sided) OR Left-Sided Heart Failure) OR Left Sided Heart Failure))

S1.3: Assessing eligibility for studies found in first search

The first search was aimed at broadly characterizing all studies that explored cocaine and cardiomyopathy and heart failure. At this level, a study was deemed relevant if it satisfied all of the following criteria: 1) The study was concerned with a relationship between cocaine with acquired cardiomyopathy of any kind and/or acquired HF of any kind; 2) The study involved human subjects; 3) The study involved clinical research in a category such as case reports, case-control studies, prospective/retrospective cohort studies, and/or randomized controlled trials.

Using the program Abstrackr,[5] three reviewers manually evaluated all 881 abstracts in randomized order to assign either "inclusion" or "exclusion" to each. Reviewers were counseled to conduct a sensitive search by keeping studies for which it was not clear from the abstract if our inclusion criteria had been met. After initial screening, 84% of the abstracts had perfect agreement between the three raters. The Fleiss inter-rater kappa, a statistical measurement of the group's agreement, was 0.51 [95% CI: 0.47, 0.55], which showed significant agreement above random chance. In addition, analysis of the Cohen inter-rater kappa between pair of users (see Figure S2-2) showed that all rater-pairs had similar agreement.[6] Forty-four of the manuscripts were automatically included after perfect agreement between the three raters. Likewise, 697 were automatically discarded.

The three reviewers met to discuss the 140 abstracts for which there were disagreements in labels. After discussion, the reviewers agreed that studies focusing on myocardial infarction, coronary dissection, coronary vasospasms, or valvular disease

were only kept if they also studied any aspect of HF or cardiomyopathy (i.e. ejection fraction, left-ventricular mass index, relative wall thickness, etc.) Any measurement modalities would be included (i.e. echocardiography, autopsy, ECG, etc.). Reviews were kept only if the abstract, or keywords, showed that cocaine and HF or cardiomyopathy were one of the main foci of the review. After the discussion, 114 of the 140 of the abstracts were approved and the remainder discarded leading to a total of 158 abstracts approved for further evaluation.

During full text evaluation, we identified 11 abstracts that were duplicates which were subsequently removed. For the remaining 147 abstracts, we first confirmed that there was a full-text associated with the abstract. Forty-one conference abstracts and poster sessions were therefore removed. Two manuscripts, after full text evaluation, were found to not fulfill the original exclusion criteria, leading to a total of 104 included manuscripts.

Next, to facilitate analysis of the 104 manuscripts, we classified each one into three exclusive categories: case reports, primary-data studies (prospective/retrospective cohort, case-control …), and reviews without primary data. A total of 24 primary data studies were obtained. Primary-data studies were further classified into association, treatment, or prognosis studies.

S1.4: Identifying hypotheses with potentially enough studies to perform a meta-analysis

The 24 primary studies identified from the first search were divided into the following groups:

- Investigation of prevalence of low LVEF in asymptomatic and symptomatic chronic cocaine users
- Acute changes in LVEF after cocaine infusion
- Cohort studies comparing LVEF in cocaine users and non-users
- Cohort studies investigating heart weight left ventricular end diastolic volume
- Wall thicknesses
- Treatment studies
- Other findings

Based on these results, we identified potentially enough studies to test the hypotheses that:

- Systolic function, as measured by LVEF, is lower in chronic cocaine users. Potentially, the prevalence of low LVEF in cocaine users is higher than non-users.
- Acute usage of cocaine results in a measurable and significant lower LVEF.
- The heart weight and wall thicknesses of cocaine users, whether measured by autopsy or by echocardiogram, is higher than those of controls.
- Similarly, the LVED is lowered in users compared to non-users.
- Beta blockers are not safe for use in chronic cocaine users.

S1.5: Terms for Second Search (Meta-analyses specific to certain outcomes and measurements): 12/21/2018

Based on these hypotheses, we conducted another search of the literature; this time with terms specific to LVEF, heart weight, LVED, and wall thickness.

Cocaine:	Heart Failure and Cardiomyopathy:
Cocaine	Heart Failure
Cocaine Hydrochloride	Cardiac Failure
Hydrochloride, Cocaine	LVEF
Cocaine HCl	left ventricular ejection fraction
HCl, Cocaine	Echocardiogram
	cardiac magnetic resonance
	Radionuclide angiography
	Cardiac MRI
	heart weight
	left ventricular mass index
	LVMI
	Relative wall thickness
	RWT
	Left ventricle size
	Left ventricular end-diastolic
	LVED
	Posterior wall thickness
	PWT

S1.6: Assessing eligibility for studies found in second search

At this level, neither case reports nor reviews were deemed eligible. Any study design beyond case-series were kept. As a result of this search, 270 additional abstracts were retrieved, and inspected in the same method as previously described. As a result fifteen additional articles were added to our review. Altogether, a total of 39 primary data studies were found and evaluated.

S1.7: Categorization of study designs

We categorized study design as follows. Studies that investigated groups of people with different exposures were categorized as cohorts, and labeled as either retrospective or prospective. Case-control categorization was limited to studies that purposefully created two groups of patients based on different health outcomes (i.e. heart failure).[7] The cross-sectional label was assigned to studies that used a one-time investigation of a study population that was not a-priori separated into groups based on risk-exposure or health outcome.

S1.8: Data extraction

For each primary data study, one researcher extracted data for each outcome and measurement modality (i.e. ejection fraction measured by echocardiogram). The following data were extracted:

- Specifics of the population (i.e. newborns, adults)
- Study design
- Sample size for each comparision group (i.e. user and non-users)
- The independent variable for cocaine use (i.e. self-reported use, urine positive test)
- Primary outcomes related to the hypotheses for the two comparison groups (i.e. ejection fraction, LVED, heart weight)

- The measurement modality for the primary outcome (i.e. echocardiography to measure LVEF, autopsy to measure heart weight, echocardiogram to measure LVMi)

Researchers were instructed any summary measures such as prevalence, odd ratios, risk ratios, and standardized mean differences. If a study did not report these effect sizes, or their confidence intervals, we attempted to extract enough primary data to calculate the effect sizes ourselves. After tables were created of extracted data, for each group of studies, one more researcher independently confirmed the accuracy of the extracted data. For each group of study design and measured outcome (Tables S3-1, S3-2 …) a summary measure was then assigned. During the meta-analysis step, the author responsible for the statistics checked each manuscript to ensure extracted data accuracy.

S1.9: Assessment of study quality

A variety of multimodal critical appraisal tools are available which assess the validity of the methodology, results, and reporting method of each study. In this review, we utilized the AXIS tool developed in a Delphi process, which evaluates cross sectional studies as well as the Downs and Black checklist, which evaluates non-randomised studies (cohort and case-control studies).[8,9] As recommended by Sanderson et al., we elected to use the appraisal tools in a checklist fashion as opposed to using specific scores.[10,11] The AXIS tool consists of 20 components -- 7 relating to the quality of reporting, 7 to study design quality, and 6 to possible introduction of biases in the study (Table S5-1). The Downs and Black checklist consists of 27 components -- 10 relating to the quality of reporting, 3 to external validity, 7 for bias, 6 for confounding, and 1 for power (Table S5-2). Additionally, we analyzed the beta-blocker treatment studies (Table S3-9) by using the Newcastle-Ottawa Rating scale.[12]

The items were used so that the reviewers would considered if a study should be discarded from the meta analysis due to low quality.

Eighteen studies were assessed for individual risk bias. These eighteen contain all studies included in the meta-analyses. Three independent authors (DJA, SB, and SZ) assessed the quality of the retrospective cohort studies using the AXIS tool. For questions for which there were disagreements between raters, we calculated the Inter-rater Fleiss kappa and permutated Cohen kappa between pairs of raters. Since the AXIS tool does not provide a numerical score for each study, the authors discussed the overall quality of each study to determine whether it would be discarded. One author (SZ) rated all 18 studies (yes = 1, no = 0) using the Downs and Black checklist to contrast the coverage of the different quality assessment tools.

For the AXIS tool, there was 100% agreement between raters in 17 of 20 questions. Disagreements were found in questions 7, 13 and 14, about concerns regarding non-responders. Because most of the studies looked retroactively at databases of autopsy reports, information about non-responders is expected to be unavailable. In addition to categorization of non-responders, the studies were consistently missing sample size justification. This was considered an important limitation since providing an *a priori* sample size calculation aims to ensure that there are enough participants in the study to yield statistically significant results. Nevertheless, the authors determined that no studies were of significantly low methodological quality; thus, no studies were discarded from the meta-analysis based on AXIS criteria.

From the Downes and Black checklist, we noticed studies were lacking descriptions about trying to blind study subjects and investigators, adjustments for different lengths of follow-up of patients, and *apriori* power analysis.

In summary, although The AXIS tool and Downs and Black checklist allowed the authors to identify a few weaknesses in the primary data studies, none of these weaknesses merited removal of any of the studies from meta-analysis.

S1.10: Data synthesis and clinical/methodological heterogeneity

The 39 primary data studies were divided into 10 groups. As some studies contained sufficient data, some were placed in multiple groups. Information for each study in each group are provided in S3 of the supplementary documents. The groups were:

- Investigation of prevalence of low LVEF in asymptomatic and symptomatic chronic cocaine users. (Tables S3-1 and S3-2)
- Acute changes in LVEF after cocaine infusion (Table S3-3)
- Cohort studies comparing LVEF in cocaine users and non-users (Tables S3-4 and S3-5)
- Cohort studies investigating heart weight (Table S3-6)
- Left ventricular end diastolic volume (LVED) (Table S3-7)
- Wall thicknesses (Table S3-8)
- Treatment studies (Table S3-9)
- Other findings (Table S3-10).

S1.11: Meta-analysis

Meta-analyses were performed for groups with more than three comparable studies. After data extraction, three authors discussed the clinical and methodological homogeneity of studies in each group to decide if the results should be mathematically pooled by the RE model. As Tables S3.1-10 show, some groups had substantive clinical and/or methodological heterogeneity. These studies would be discussed individually.

S1.12: Statistical heterogeneity from meta-analysis and assessment of publication bias

For each meta-analysis, heterogeneity across studies was calculated by first calculating the total variance (Q), the degrees of freedom (df), and the I^2 statistic.[13] . Prevalence meta-analysis was performed using the double arcsine transformation. This is the recommended transformation for combining proportions close to 0 or 1.[14] Assessment of risk across publications was explored using funnel plots and two tests: the rank correlation and the regression test for funnel plot asymmetry.[15,16] However, it should be emphasized that publication bias tests have very low statistical power to detect a positive unless the sample sizes are large.[17,18]

S1.13: Sub-group analysis

Prior to meta-analyses, the authors decided that subgroup analysis would be performed for each meta-analysis in which sufficient studies were available to create subgroups with more than two studies each.

References:

1. Dickersin, K., Scherer, R. & Lefebvre, C. Systematic reviews: identifying relevant studies for systematic reviews. *Bmj* **309**, 1286–1291 (1994).
2. Doust, J. A., Pietrzak, E., Dobson, A. & Glasziou, P. How well does B-type natriuretic peptide predict death and cardiac events in patients with heart failure: systematic review. *Bmj* **330**, 625 (2005).
3. Smith, G. L. *et al.* Renal impairment and outcomes in heart failure: systematic review and meta-analysis. *J. Am. Coll. Cardiol.* **47**, 1987–1996 (2006).
4. van der Linde, D. *et al.* Birth prevalence of congenital heart disease worldwide: a systematic review and meta-analysis. *J. Am. Coll. Cardiol.* **58**, 2241–2247 (2011).

5. Wallace, B. C., Small, K., Brodley, C. E., Lau, J. & Trikalinos, T. A. Deploying an interactive machine learning system in an evidence-based practice center: abstrackr. in *proceedings of the 2nd ACM SIGHIT International Health Informatics Symposium* 819–824 (ACM, 2012).
6. Arenas, D. J. Inter-Rater: Software for analysis of inter-rater reliability by permutating pairs of multiple users. *ArXiv Prepr. ArXiv180905731* (2018).
7. Lewallen, S. & Courtright, P. Epidemiology in practice: case-control studies. *Community Eye Health* **11**, 57 (1998).
8. Downes, M. J., Brennan, M. L., Williams, H. C. & Dean, R. S. Development of a critical appraisal tool to assess the quality of cross-sectional studies (AXIS). *BMJ Open* **6**, e011458 (2016).
9. Downs, S. H. & Black, N. The feasibility of creating a checklist for the assessment of the methodological quality both of randomised and non-randomised studies of health care interventions. *J. Epidemiol. Community Health* **52**, 377–384 (1998).
10. Sanderson, S., Tatt, I. D. & Higgins, J. Tools for assessing quality and susceptibility to bias in observational studies in epidemiology: a systematic review and annotated bibliography. *Int. J. Epidemiol.* **36**, 666–676 (2007).
11. Rao, G. *et al.* Methodological standards for meta-analyses and qualitative systematic reviews of cardiac prevention and treatment studies: a scientific statement from the American Heart Association. *Circulation* **136**, e172–e194 (2017).
12. Wells, G. *et al. Newcastle-Ottawa quality assessment scale cohort studies*. (2014).
13. Higgins, J. P. & Thompson, S. G. Quantifying heterogeneity in a meta-analysis. *Stat. Med.* **21**, 1539–1558 (2002).
14. Barendregt, J. J., Doi, S. A., Lee, Y. Y., Norman, R. E. & Vos, T. Meta-analysis of prevalence. *J Epidemiol Community Health* jech–2013 (2013).

15. Begg, C. B. & Mazumdar, M. Operating characteristics of a rank correlation test for publication bias. *Biometrics* 1088–1101 (1994).

16. Egger, M., Smith, G. D., Schneider, M. & Minder, C. Bias in meta-analysis detected by a simple, graphical test. *Bmj* **315**, 629–634 (1997).

17. Sterne, J. A., Gavaghan, D. & Egger, M. Publication and related bias in meta-analysis: power of statistical tests and prevalence in the literature. *J. Clin. Epidemiol.* **53**, 1119–1129 (2000).

18. Deeks, J. J., Macaskill, P. & Irwig, L. The performance of tests of publication bias and other sample size effects in systematic reviews of diagnostic test accuracy was assessed. *J. Clin. Epidemiol.* **58**, 882–893 (2005).
