# Supplementary material for: Cocaine, cardiomyopathy, and heart failure: a systematic review and meta-analysis
Source: Sci Rep. 2020 Nov 13;10:19795. doi: 10.1038/s41598-020-76273-1 (PMC7666138; doi:10.1038/s41598-020-76273-1)
Supplement: Supplementary file 2 — Supplementary Information 2. [file 41598_2020_76273_MOESM2_ESM.pdf]

Cocaine, cardiomyopathy, and heart failure: A systematic review and meta-analysis

Supplementary Document S2:
Additional Figures

Daniel J. Arenas, PhD[a]
Sourik Beltran, BA[a,b]
Sara Zhou, BA[c]
Lee R. Goldberg, MD, MPH, FACC[a]

[a]Perelman School of Medicine at the University of Pennsylvania
[b]Department of Medical Ethics and Health Policy, University of Pennsylvania
[c]College of Arts and Sciences, University of Pennsylvania

Corresponding Author:
Lee R. Goldberg, MD, MPH, FACC
Penn Medicine Heart Failure and Cardiac Transplant Center
11-171 South Tower, Perelman Center for Advanced Medicine
3400 Civic Center Blvd
Philadelphia PA, 19104
Office: (215) 615-0820
Fax: (215) 615-0828
Email: Lee.Goldberg@pennmedicine.upenn.edu

OTHER AUTHOR CONTACT INFORMATION:
Daniel J. Arenas: Daniel.Arenas@pennmedicine.upenn.edu
Sourik Beltran: Sourik.Beltran@pennmedicine.upenn.edu
Sara Zhou: sarazhou@sas.upenn.edu

Figure S2.1 Overview of search and inclusion methodology.

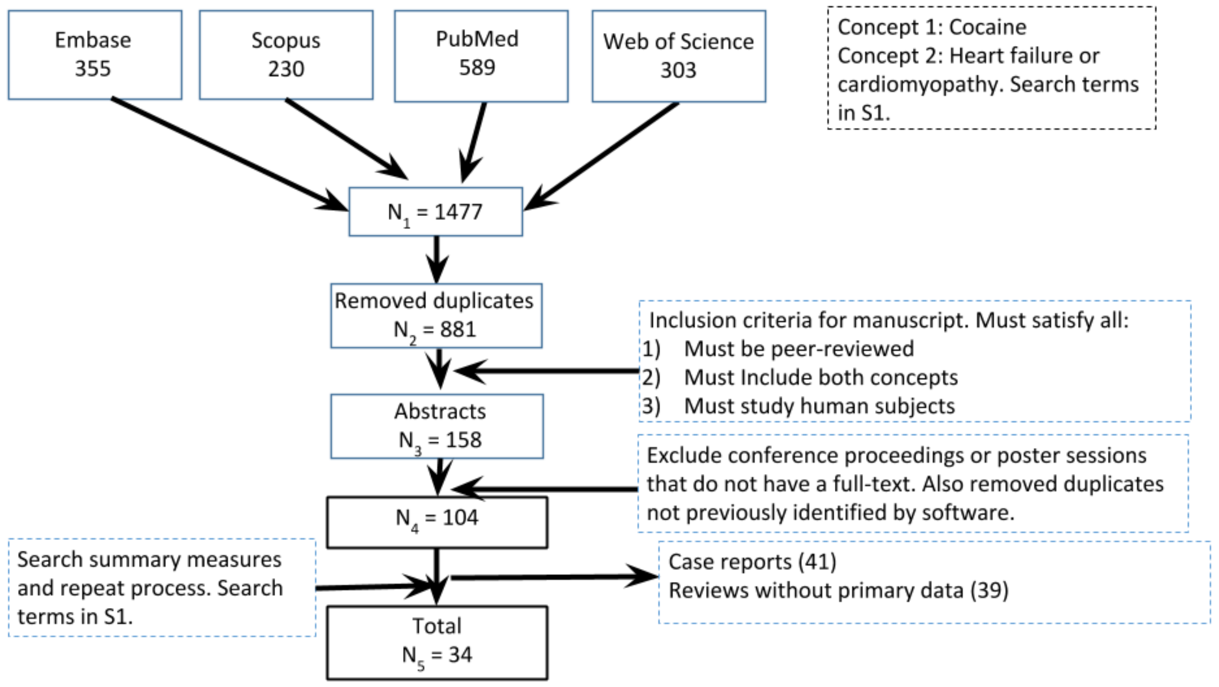

Figure S2-2. Inter-rater reliability for the inclusion/exclusion of abstracts.

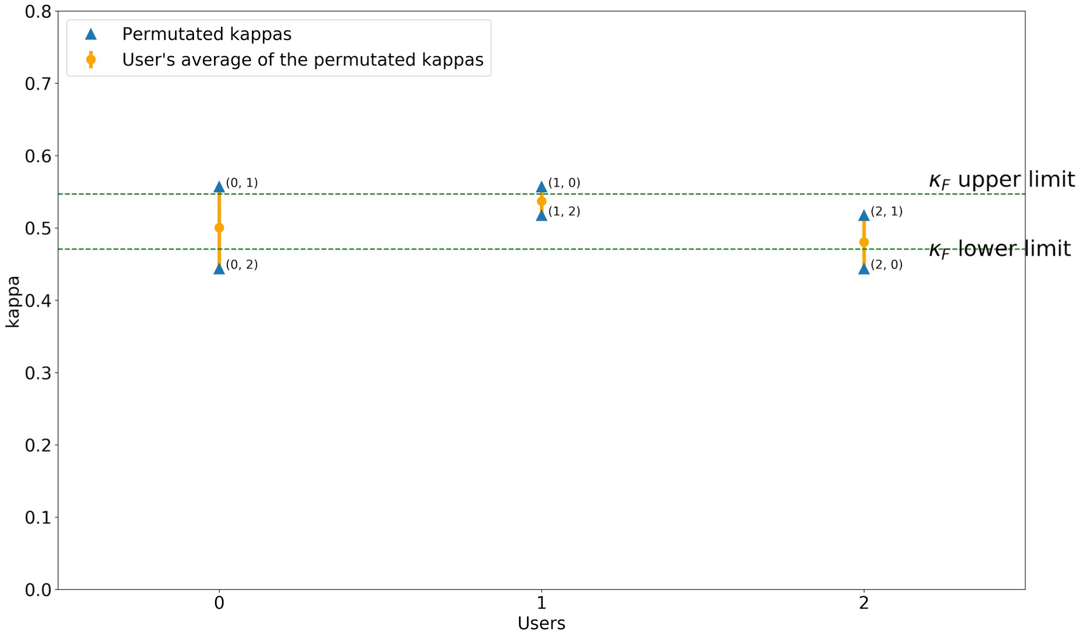

Three users (0, 1, and 2) reviewed 881 abstracts. The Cohen kappa, reliability between two raters, is shown in blue triangles for permutation of different pairs of users (i.e. 0 with 1, 0 with 2, etc.). For each user the average and confidence interval of the permutations is shown in orange. Green lines delimit the 95% confidence interval for the Fleiss inter-rater kappa, the overall group agreement. The results show that the group's agreement is significantly above random chance, and that no user was statistically lower in agreement than the other users. A copy and manual of the software used for the analysis of inter-rater reliability can be found in reference[1].

Figure S2-3. Prevalence. Meta-analysis of studies investigating the prevalence of low-EF in asymptomatic chronic cocaine users.

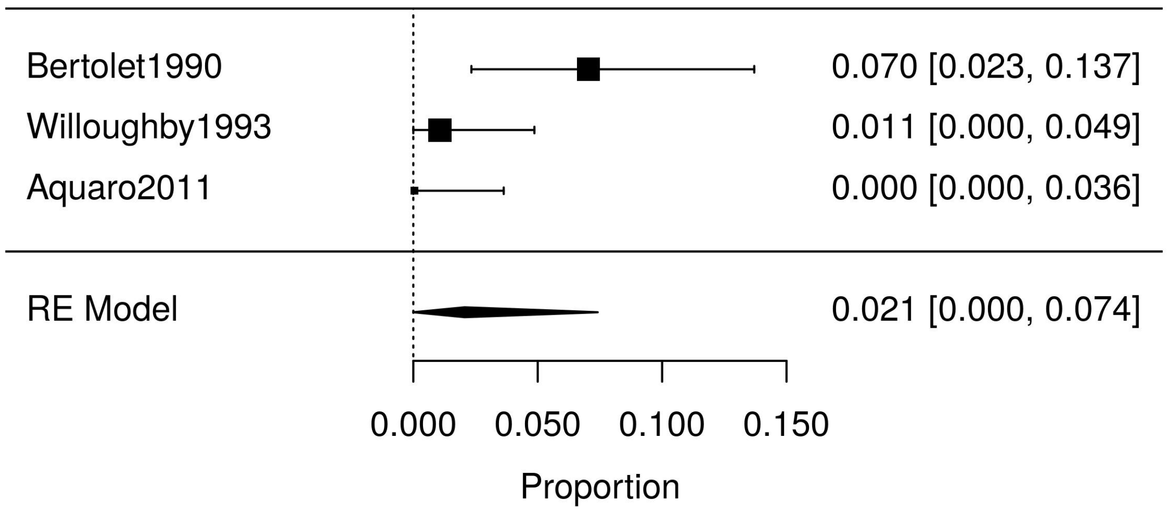

Figure S2-4. LV Function. Meta-analysis of studies investigating the effect of chronic cocaine use on LV function if we remove the study by Maceira et al. which did not use previous cardiovascular disease as an exclusion criterion.

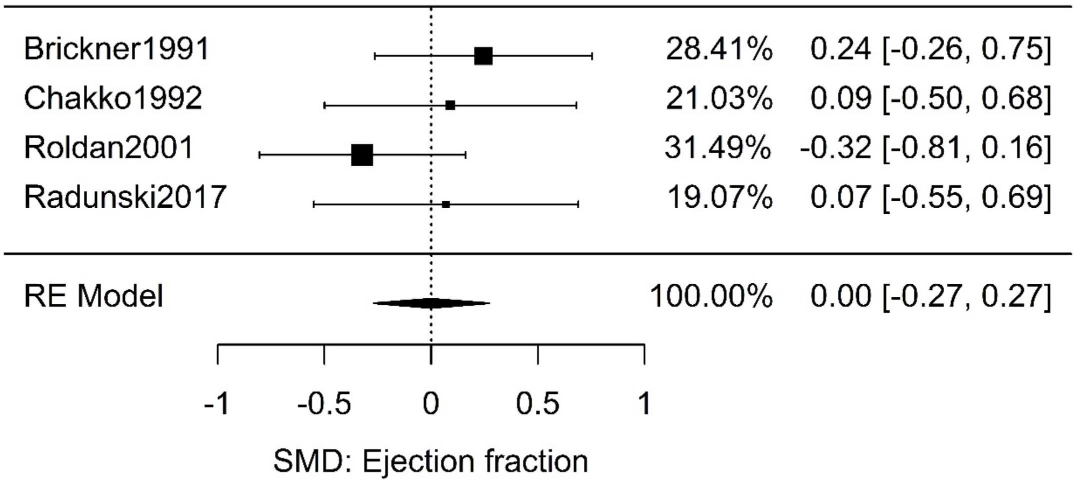

Figure S2-5. Subgroup analyses of studies investigating the effect of chronic cocaine use on heart weight: Four autopsy and seven echocardiogram studies.

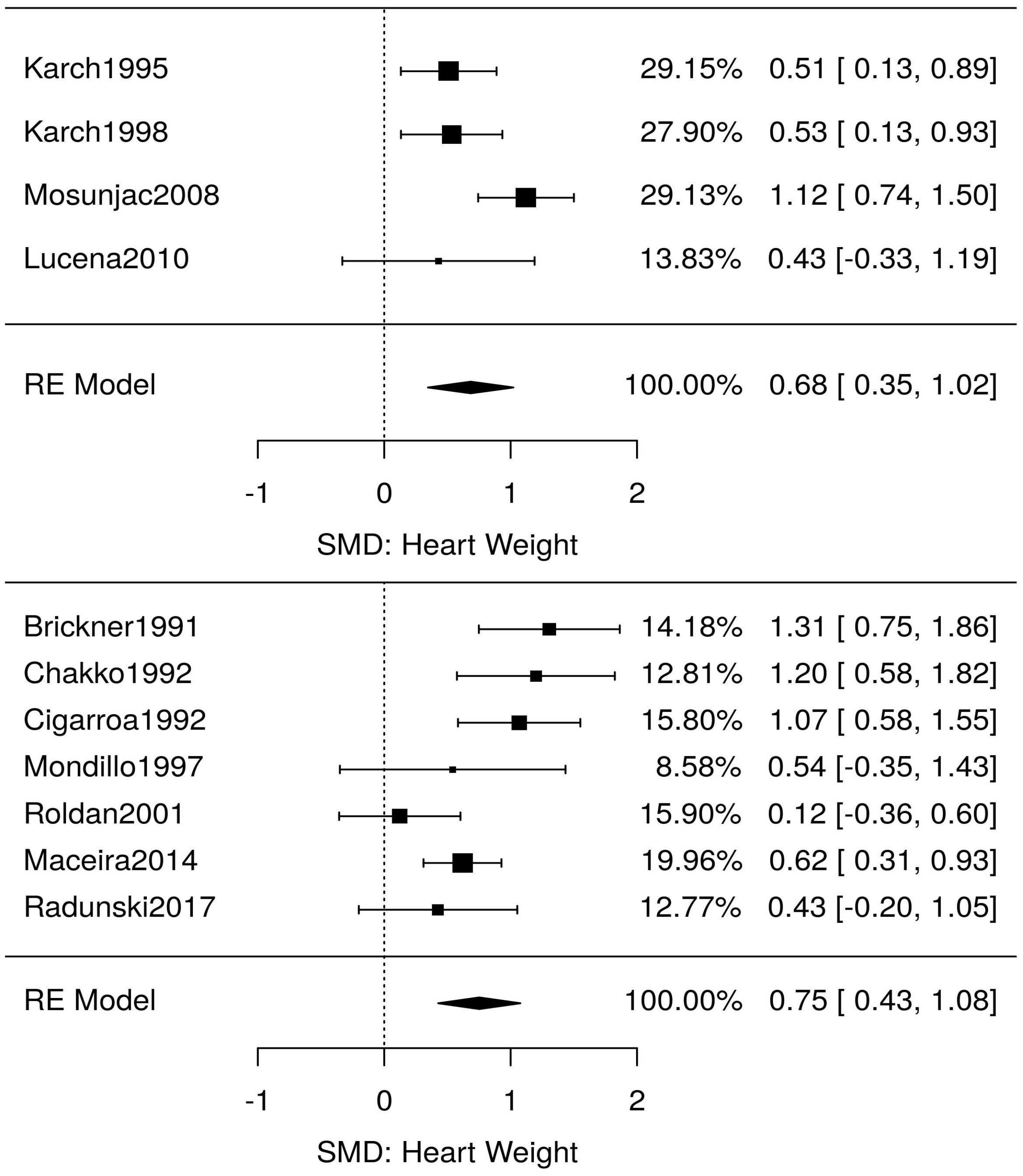

Figure S2-6. Fractional shortening. Meta-analysis of studies investigating the effect of chronic cocaine use on fractional shortening (FS).

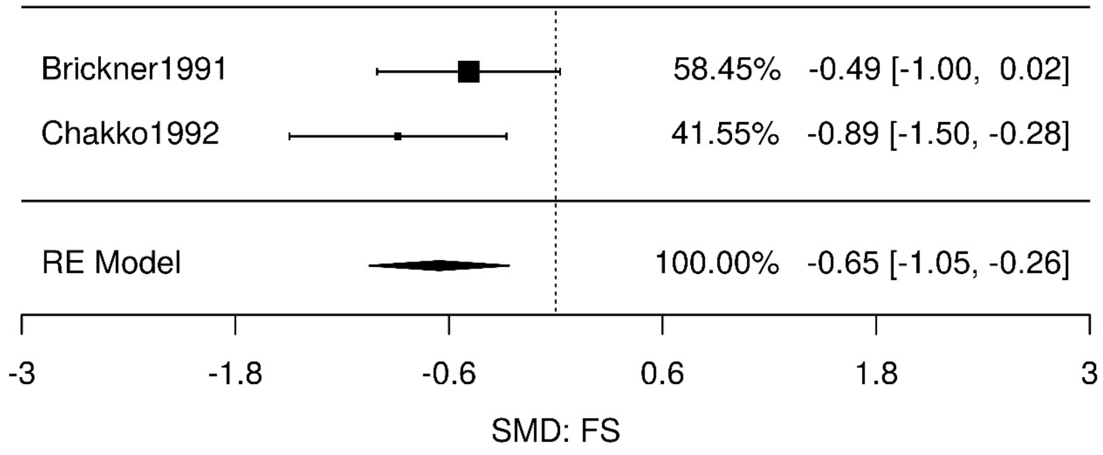

Figure S2-7. E/A Ratio. Meta-analysis of studies investigating the effect of chronic cocaine use on fractional shortening (FS).

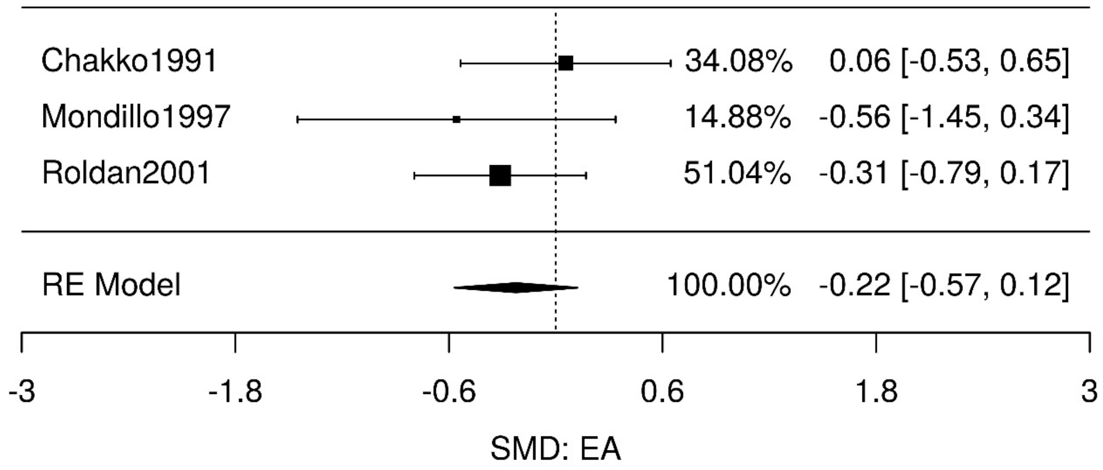

Figure S2-8. Funnel plots for calculated effects size of cocaine on ejection fraction and heart weight.

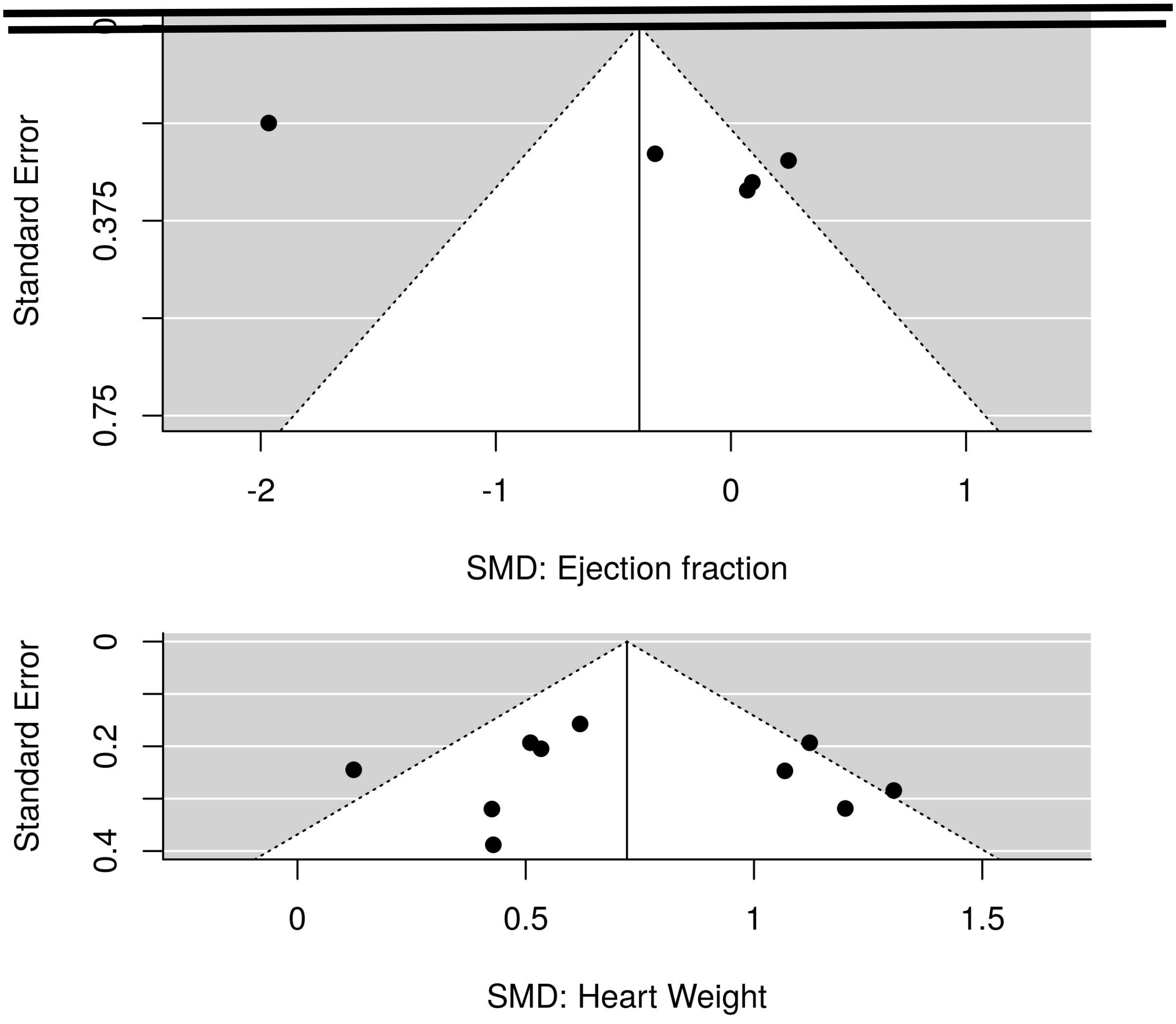

The funnel plot explores publication bias by plotting the effect sizes versus a measure of variance. The main concept is that if there is no publication bias, then studies with high variance (lower in the y-axis) should be symmetrically distributed across the average value. The Rank Correlation and Regression tests, which quantify the asymmetry, did not suggest publication bias.

Figure S2-9. Funnel plots for calculated effects size of cocaine on LVED and wall thickness.

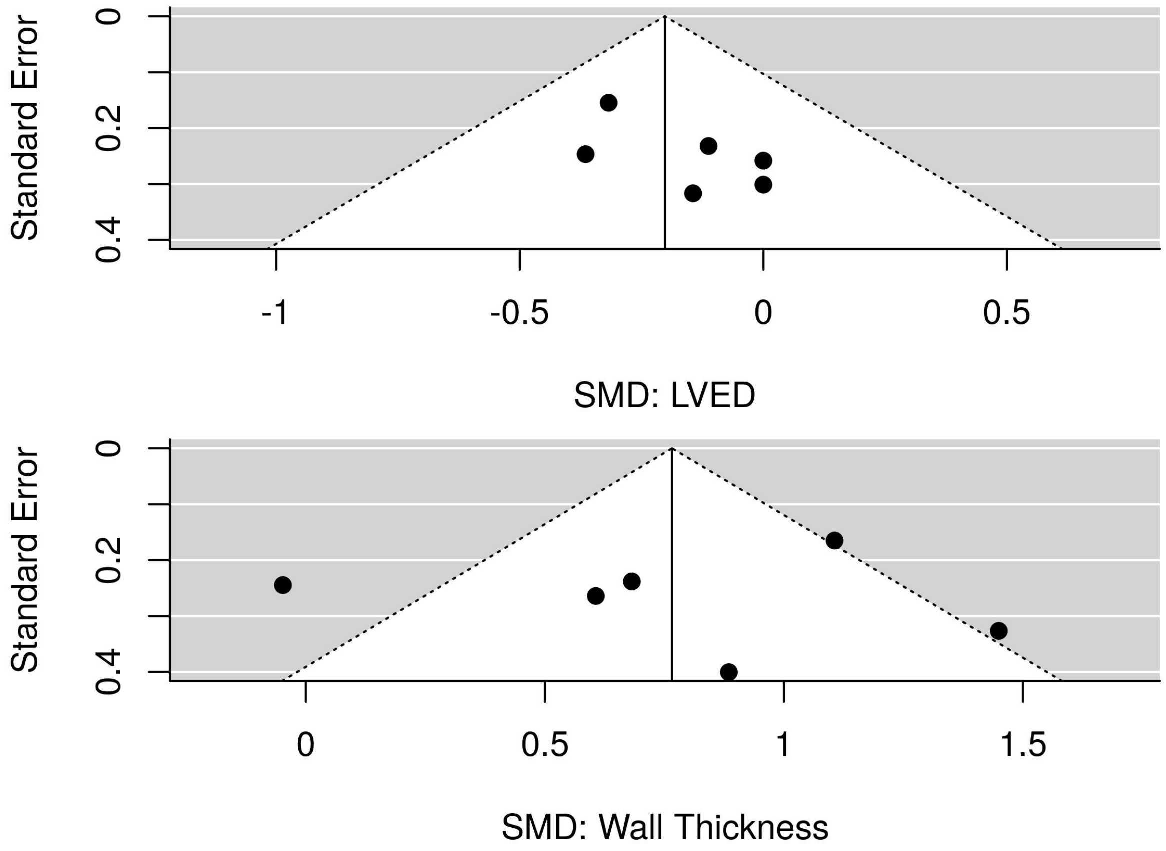

References:

1. Arenas, D. J. Inter-Rater: Software for analysis of inter-rater reliability by permutating pairs of multiple users. *ArXiv Prepr. ArXiv180905731* (2018).
