# Supplementary material for: Cocaine, cardiomyopathy, and heart failure: a systematic review and meta-analysis
Source: Sci Rep. 2020 Nov 13;10:19795. doi: 10.1038/s41598-020-76273-1 (PMC7666138; doi:10.1038/s41598-020-76273-1)
Supplement: Supplementary file 3 — Supplementary Information 3. [file 41598_2020_76273_MOESM3_ESM.pdf]

Cocaine, cardiomyopathy, and heart failure: A systematic review and meta-analysis

Supplementary Document S3:
Data Tables

Daniel J. Arenas, PhD[a]
Sourik Beltran, BA[a,b]
Sara Zhou, BA[c]
Lee R. Goldberg, MD, MPH, FACC[a]

[a]Perelman School of Medicine at the University of Pennsylvania
[b]Department of Medical Ethics and Health Policy, University of Pennsylvania
[c]College of Arts and Sciences, University of Pennsylvania

Corresponding Author:
Lee R. Goldberg, MD, MPH, FACC
Penn Medicine Heart Failure and Cardiac Transplant Center
11-171 South Tower, Perelman Center for Advanced Medicine
3400 Civic Center Blvd
Philadelphia PA, 19104
Office: (215) 615-0820
Fax: (215) 615-0828
Email: Lee.Goldberg@pennmedicine.upenn.edu

OTHER AUTHOR CONTACT INFORMATION:
Daniel J. Arenas: Daniel.Arenas@pennmedicine.upenn.edu
Sourik Beltran: Sourik.Beltran@pennmedicine.upenn.edu
Sara Zhou: sarazhou@sas.upenn.edu

Table S3-1. Studies with cross-sectional data investigating prevalence of low EF in asymptomatic cocaine users.

Study	Subjects	*N*	Cocaine use measured by:	EF measured by:	Summary measure: Prevalence
Bertolet et al. 1990[1]	Asymptomatic-male users. Age = 36. Range (25-44)	84	Urine toxicology	Radionuclide angiography (LVEF < 50%)	0.070 [0.023, 0.137] **: Second paragraph of Results*
Willoughby et al. 1993[2]	Asymptomatic Adults with HIV and drug use. Age = 36 +/- 7	85	Self-reported	Echocardiogram (LVEF < 50%)	0.012 [0.000, 0.049] **: Page 2, fourth paragraph*
Aquaro et al. 2011[3]	Asymptomatic users. Age = 39 +/- 7 years	50	Urine toxicology	CMR (LVEF < 55%)	0.000 [0.000, 0.036] **: Page 3, 7th paragraph*

*: Denotes that the summary measure was calculated by writers of this systematic review. The location of the primary data is then reported.

RMRS: Retrospective medical records search.

CMR: Cardio magnetic resonance.

Table S3-2. Studies with cross-sectional data investigating prevalence of low EF in symptomatic cocaine users.

**Study**	**Subjects**	***N***	**Cocaine use measured by:**	**EF measured by:**	**Prevalence:**
Om et al. 1992[4]	Adults with recent cocaine use requiring a coronary angiogram.	33	Urine toxicology	Echocardiogram<br>LVEF < 50%	0.545 [0.372, 0.713]<br>**: 6th paragraph of Results*
HollanderAIM et al. 1995[5]	Adults with recent cocaine use who suffered MI in hospital	122	RMRS	RMRS: Mentions of heart failure	0.055 [0.020, 0.105]<br>**: Table 4*
Weber et al. 2000[6]	Adults presenting to ED with chest discomfort and recent cocaine use	196	RMRS	RMRS: Mentions of heart failure	0.020 [0.004, 0.271]<br>**: Table 1*

*: Denotes that the summary measure was calculated by writers of this systematic review. The location of the primary data is then reported. The location of the primary data is then reported.
RMRS: Retrospective medical records search

The type of chest complaint varied greatly between studies; therefore, meta-analysis was deemed inappropriate.

Table S3-3. Studies exploring acute changes in LVEF after cocaine infusion

Study	Study characteristics	Important findings:
Eisenberg et al. 1993[7]	Intravenous cocaine infusion (n = 14).<br><br>Placebo (n = 2).	No significant changes in LVEF.
Pitts et al. 1998[8]	Infusion by catheterization (n = 20).<br><br>Same patients used as control when infused with saline. (n= 20).	Significant drop in LVEF fifteen minutes after cocaine infusion

Table S3-4. Cohort studies investigating EF in asymptomatic chronic cocaine users.

Study	Cohorts	EF measured by:	Summary measures:
Brickner et al. 1991[9]	1: Long-term asymptomatic cocaine users. (N_1 = 30)<br>2: Aged and race matched. (N_2 = 30)	Echocardiogram: Fractional shortening	g = + 0.24 [-0.26, 0.75]]<br>*: *Table 1*
Chakko et al. 1992[10]	1: Long-term asymptomatic cocaine users. (N_1 = 52)<br>2: Aged match. (N_2 = 14)	Echocardiogram	g = + 0.09 [-0.50, 0.68]<br>*: *Table 2*
Roldan et al. 2001[11]	1: Long-term asymptomatic cocaine users. (N_1= 35)<br>2: Agematch. (N_2 = 32)	Echocardiogram	g_{rest} = - 0.32 [-0.81, 0.16]<br>*: *Table 2*
Maceira 2014 et al.[12]	1: Asymptomatic long term cocaine users. (N_1 = 94)<br>2: Age and gender matched non-users. (N_2 = 80)	Cardiovascular MRI	g = - 1.97 [-2.33, -1.60<br>*: *Table 5*
Radunski et al. 2017[13]	1: Asymptomatic long-term cocaine users. (N_1 = 20)<br>2: Non-users age matched. (N_2 = 20)	Cardiovascular MRI	g = 0.07 [-0.55, 069]<br>*,@: *Table 4.*
Tong et al. 2004[14]	1: Long-term cocaine users. (N_1 = 123)<br>2: Non-users. Not age-matched. (N_2 = 15)	Echocardiogram	g = - 0.30 [-0.84, 0.23]<br>*: *Table 2*

*: Denotes that the summary measure was calculated by writers of this systematic review. The location of the primary data is then reported.

@: Median was reported, not the average. Interquartile range reported, not the standard deviation. Median and SD back-calculated from normal-distribution assumption

Table S3-5. Cohort studies investigating heart failure in cocaine users presenting to ED with chest complaints.

Study	Cohorts	EF measured by:	Summary measures:
Carrillo et al. 2011[15]	1: Long-term cocaine users admitted for ACS. (N_1= 24)<br>2: Non-cocaine users admitted for ACS. (N_2= 379)	Echocardiogram	g = - 0.61 [-1.02, -0.19]<br>*: *Table 2*
Frustaci et al. 2015[16]	1: Symptomatic cocaine users. (N_1 = 10)<br>2: Matched group. (N_2 = 10)	Cardiovascular MRI	g = - 3.65 [-5.05, -2.22]<br>*: *Table 1*
Dierck et al. 2008[17]	Patients presenting to ED with heart failure<br>1: Cocaine or methamphetamine use (N_1 = 594)<br>2: Non-users. Not age-matched. (N_2 = 10,664)	Chart review. Methods not reported	*OR* = 3.4 [2.8, 4.2]<br>*Table 1*

Due to limited data and differences in each study's methodology, meta-analysis was deemed inappropriate.

Table S3-6. Cohort studies investigating heart weight by autopsy or echocardiogram.

Study	Cohorts	Heart Weight measured by:	Summary measures
Karch et al. 1995[18!]	1: Trauma fatalities, testing (+) for cocaine (N_1 = 32). Men Only<br>2: Matched group, (-) for cocaine (N_2 = 51)	Autopsy	*g* = + 0.48 [0.037, 0.924]<br>**: 3rd paragraph of results*
Karch et al. 1995[18!]	1: Trauma fatalities, testing (+) for cocaine (N = 16). Women Only.<br>2: Matched group, (-) for cocaine (N = 16).	Autopsy	*g* = + 0.59 [-0.11, 1.28]<br>**: 3rd paragraph of results*
Karch et al. 1995[18!]	Combination by Random Effect Model		*g* = 0.51 [0.13, 0.89]
Karch et al. 1998[19!!]	1: Cocaine related deaths. (N_1 = 48)<br>2: Matched group, trauma death, (-) for cocaine. (N_2 = 51)	Autopsy	*g* = + 0.53 [0.13, 0.93]<br>**: 2nd paragraph of findings*
Mosunjac et al. 2008[20]	1: Acute or chronic cocaine use (N_1 = 63)<br>2: Matched group, (-) non-users (N_2 = 61)	Autopsy	*g* = + 1.12 [0.74, 1.50]<br>**: 3rd paragraph of results*
Lucena et al. 2010[21]	1: Cocaine-related deaths. (N_1 = 21)<br>2: Matched group. (N_2 = 10)	Autopsy	*g* = + 0.43 [-0.33, 1.19]<br>**: 4th paragraph of methods and cardiovascular findings section of results*
Brickner et al. 1991[9]	1: Long-term asymptomatic cocaine users . (N_1 = 30)<br>2: Aged and race matched. (N_2 = 30)	Echocardiogram: LVMi	*g* = + 1.31 [0.75, 1.86]<br>**: Table 1*
Cigarroa et al. 1992[22]	1: Asymptomatic male chronic cocaine users. (N_1 = 49)<br>2: Age and race-matched group (N = 30)	Echocardiogram: LVMi	*g* = + 1.07 [0.58, 1.55]<br>**: Table 1. Combined cocaine using groups*
Chakko et al. 1992[10]	1: Chronic cocaine users in rehabilitation (N = 52)<br>2: Matched group, (+) for alcohol and smoking use	Echocardiogram: LVMi	*g* = + 1.50 [0.86, 2.15]<br>**: Table 2*

	(N = 14)		
Mondillo et al. 1997[23]	1: Chronic cocaine users (N = 10)<br>2: Non-users. Matched for age. (N = 10)	Echocardiogram: LVMi	*g* = + *0.54 [-0.35, 1.43]*<br>*: *Table 1*
Roldan et al. 2001[11]	1: Asymptomatic chronic cocaine user (N = 35).<br>2: Aged-matched group (N = 32).	Echocardiogram: LVMi	*g* = + 0.00 [-0.48, 0.48]<br>*: *Table 2*
Maceira et al. 2014[12]	1: Asymptomatic long term cocaine users (N_1 = 94)<br>2: Age and gender matched non-users (N_2 = 80)	Echocardiogram: LVMi	*g* = + 0.62 [0.31, 0.93]<br>*: *Table 5*
Radunski et al. 2017[13]	1: Asymptomatic long-term cocaine users (N_1 = 20)<br>2: Non-users. Aged-matched group. (N_2 = 20)	Cardiovascular MRI: LVMi	*g* = + 0.43 [-0.20, 1.05]<br>*,@: *Table 4*
Fineschi et al. 1997[24]	1: Cocaine associated death. (N_1 = 26).<br>2: Head trauma death.<br>(Age difference is statistically significant)[t]<br>(N_2 = 26)	Autopsy	*: *Table 1*
Tong et al. 2004[14]	1: Asymptomatic chronic cocaine users (N = 123)<br>2: Non-users. (Age difference statistically significant)[P]<br>(N = 15)	Echocardiogram: LVMi	*: *Table 2*
Fineschi et al. 2005[25]	1: Deaths attributed to cocaine abuse (N_1 = 26)<br>2: Accidental death. (Age difference is statistically significant)[t] (N_2 = 21)	Autopsy	*:*Table 1*

!: Karch1995 study. Clark County Coroner Office, Nevada. 1993-1994.

!!: Karch1998 study. San Francisco Medical Examiner 1989-1995

t: Statistical significance by *t*-test.

P: Per original authors

*: Denotes that the summary measure was calculated by writers of this systematic review. The location of the primary data is then reported.

@: Median was reported, not the average. Interquartile range reported, not the standard deviation. Median and SD back-calculated from normal-distribution assumption.

Table S3-7. Cohort studies investigating Left Ventricular End Diastolic Volume.

Study	Cohorts	LVED measured by:	Summary measures
Brickner et al. 1991[9]	1: Long-term asymptomatic cocaine users (N_1 = 30)<br>2: Aged and race matched (N_2 = 30)	Echocardiogram: LVED	*g* = + 0.00 [-0.51, 0.51]<br>**: Table 1*
Chakko et al. 1992[10]	1: Chronic cocaine users in rehabilitation (N_1 = 52)<br>2: Matched group, (+) for alcohol and smoking use (N_2 = 14)	Echocardiogram: LVED	*g* = 0.00 [-0.59, 0.59]<br>**: Table 2*
Cigarroa et al. 1992[22]	1: Asymptomatic male chronic cocaine users (N_1 = 49)<br>2: Age and race-matched group (N_2 = 30)	Echocardiogram: LVED	*g* = -0.11 [-0.57, 0.34]<br>**: Table 1. Combined cocaine using groups*
Roldan et al. 2001[11]	1: Asymptomatic chronic cocaine user (N_1 = 35)<br>2: Aged-matched group (N_2 = 32)	Echocardiogram: LVED	*g* = - 0.36 [-0.85, 0.12]<br>**: Table 2*
Maceira et al. 2014[12]	1: Asymptomatic long term cocaine users (N_1 = 94)<br>2: Age and gender matched non-users (N_2 = 80)	Echocardiogram: LVED	g = - 0.32 [-0.62, -0.01]<br>**: Table 5*
Radunski et al. 2017[13]	1: Asymptomatic long-term cocaine users (N_1 = 20)<br>2: Non-users (N_2 = 20)	Cardiovascular MRI: LVED	*g* = - 0.14 [-0.76, 0.48]<br>**,@: Table 4*

**:* Denotes that the summary measure was calculated by writers of this systematic review. The location of the primary data is then reported.

@: Median was reported, not the average. Interquartile range reported, not the standard deviation. Median and SD back-calculated from normal-distribution assumption

Table S3-8. Cohort studies investigating Total and Relative Wall Thickness.

Study	Cohorts	Thickness measured by:	Summary measures
Brickner et al. 1991[9]	1: Long-term asymptomatic cocaine users (N_1 = 30)<br>2: Aged and race matched (N_2 = 30)	Echocardiogram. RWT calculated using PWT and LVDD.[Bierig2005]	*g* = + 0.61 [0.09, 1.12]<br>**: Table 1*
Chakko et al. 1992[10]	1: Chronic cocaine users in rehabilitation (N_1 = 52)<br>2: Matched group, (+) for alcohol and smoking use (N_2 = 14)	Echocardiogram. RWT calculated using PWT and LVDD.[Bierig2005]	*g* = + 1.45 [0.81, 2.09]<br>**: Table 2*
Cigarroa et al. 1992[22]	1: Asymptomatic male chronic cocaine users (N_1 = 49)<br>2: Age and race-matched group (N_2 = 30)	Echocardiogram. RWT calculated using PWT and LVDD	*g* = + 0.68 [0.22, 1.15]<br>**: Table 1. Combined cocaine using groups*
Mosunjac et al. 2008[20]	1: Acute or chronic cocaine use (N_1 = 63)<br>2: Matched group, (-) for acute or chronic cocaine use (N_2 = 61)	Autopsy. Presence of LVH defined as PWT > 1.2 cm and heart weight > 450 g	*RR* = 1.61 [1.15, 2.37]<br>**: 2nd paragraph of Results*
Lucena et al. 2010[21]	1: Cocaine-related deaths (N_1 = 21)<br>2: Marched group (N_2 = 10)	Autopsy. LV wall thickness	*g* = + 0.88 [0.10, 1.67]<br>**: 6th paragraphs of results*
Roldan et al. 2001[11]	1: Asymptomatic chronic cocaine user (N_1 = 35)<br>2: Aged-matched group (N_2 = 32)	Echocardiogram. RWT calculated using PWT and LVDD	*g* = - 0.05 [-0.53, 0.43]<br>**: Table 2*
Maceira et al. 2014[12]	1: Asymptomatic long term cocaine users (N_1 = 94)<br>2: Age and gender matched non-users (N_2 = 80)	Echocardiogram. Relative wall mass	*g* = + 1.11 [0.78, 1.43]<br>**: Table 5*

The study by Mosunjac had sufficient data to calculate the risk-ratio of left ventricular hypertrophy between the two groups (but not enough to calculate other effect sizes).

Table S3-9. Retrospective studies investigating treatment of cocaine users with cardiac complications. All studies in United States.

Study	Subject Group	Comparison Group	Total no. of participants	Exclude ACS patients?	Study period (years)	Average treatment/follow-up (months)	Primary disease outcome(s)	Other outcomes
Nguyen et al., 2017	ADHF patients with **positive** history of chronic cocaine use	ADFH patients with **no** history of cocaine use	90 vs 177	Yes	19	32 months	Readmission for ADHF, Hypertensive Emergency, Stroke, ACS, Death	None
""	[illegible] with **positive** history of chronic cocaine use. Treated [illegible]	patients with **positive** history of chronic cocaine use. Treated with [illegible]	54 vs 36	""	""	""	Mortality	""
Egbuche et al., 2018	HFrEF patients positive for cocaine. **Treated [illegible]**	[illegible] patients positive for cocaine. **Treated with other medications**	231 vs 37	No	3.92	12 months	30-day readmission due to all-cause and heart failure	1-year mortality rate
Lopez et al., 2019	[illegible] patients with active cocaine use. **Treated [illegible]**	[illegible] patients with active cocaine use. **Treated with other medications**	38 vs 34	No	1	12 months	NYHA Functional Classification	LVEF

Table S3-10. Other findings.

Study	Patients	Important findings
Kasper et al. 1994[30]	Patients admitted for heart failure due to dilated cardiomyopathy. (n = 673).	About 1% of dilated cardiomyopathy cases were related to cocaine use.
Willet et al. 1995[31]	Asymptomatic chronic cocaine users.<br>Blacks (n = 79)<br>Whites (n = 33)	LV mass, posterior and septal wall thickness were significantly higher in Black chronic cocaine users.
Melon et al. 1997[32]	Asymptomatic chronic cocaine users. (n = 9).<br>Asymptomatic non-users. (n = 6).	Radio-chemical study by C-11-hydroxyephedrine demonstrates reduced NE reuptake in cocaine users, which suggests prolonged exposure to elevated catecholamine levels.
Mehta et al. 2002[33]	Newborns exposed to cocaine in utero (n = 82).<br>Normal controls (n = 87).	Prenatal exposure to cocaine results in regional wall abnormalities but no significant difference in systolic function.

The study by Willett et al. of cocaine users directly compared septal wall and posterior wall thicknesses between whites and blacks and found significantly higher values for the latter.[31] Since there were no comparison to non-cocaine users, the data could not be included in the meta-analysism of relative wall thickness.

Only one manuscript was identified which studied systolic function among newborns exposed to cocaine in utero which found zero of the 82 patients included in the study were found to have a low LVEF.[33]

We did not identify any articles which investigated clinical prognosis of HF in chronic cocaine users.

Table S3-11. Results from heterogeneity and publication bias tests.

**Meta-analysis**	**Number of studies**	***Q***	***I*²**	**Rank correlation test. Kendall's *τ*:**	***p-value***	**Regression test. *z:***	***p-value***
Prevalence. Low EF. Asymptomatic	3	6.6	69.8	-0.33	1.00	-0.95	0.34
Ejection Fraction. Asymptomatic.	5	74.3	94.6	0.40	0.48	2.89	0.00
Heart Weight	11	22.7	55.9	0.09	0.76	-0.05	0.96
LVED. Asymptomatic.	6	2.2	0.0	0.33	0.47	1.00	0.32
RWT. Asymptomatic.	5	20.2	80.2	-0.07	1.00	0.31	0.76
**SubGroup (Heart Weight)**							
Autopsy	4	6.88	56.4	0.33	0.75	-0.61	0.54
LVMi	7	15.9	62.3	0.14	0.77	0.16	0.88

References:

1. Bertolet, B. D. *et al.* Unrecognized left ventricular dysfunction in an apparently healthy cocaine abuse population. *Clin. Cardiol.* **13**, 323–328 (1990).
2. Willoughby, S. B., Vlahov, D. & Herskowitz, A. Frequency of left ventricular dysfunction and other echocardiographic abnormalities in human immunodeficiency virus seronegative intravenous drug users. *Am. J. Cardiol.* **71**, 446–447 (1993).
3. Aquaro, G. D. *et al.* Silent myocardial damage in cocaine addicts. *Heart* hrt–2011 (2011).
4. Om, A. Cardiovascular complications of cocaine. *Am. J. Med. Sci.* **303**, 333–339 (1992).
5. Hollander, J. E. *et al.* Cocaine-associated myocardial infarction: mortality and complications. *Arch. Intern. Med.* **155**, 1081–1086 (1995).
6. Weber, J. E. *et al.* Cocaine-associated Chest Pain How Common Is Myocardial Infarction? *Acad. Emerg. Med.* **7**, 873–877 (2000).
7. Eisenberg, M. J. *et al.* Left ventricular function immediately after intravenous cocaine: a quantitative two-dimensional echocardiographic study. *J. Am. Coll. Cardiol.* **22**, 1581–1586 (1993).
8. Pitts, W. R., Vongpatanasin, W., Cigarroa, J. E., Hillis, L. D. & Lange, R. A. Effects of the intracoronary infusion of cocaine on left ventricular systolic and diastolic function in humans. *Circulation* **97**, 1270–1273 (1998).
9. Brickner, M. E., Willard, J. E., Eichhorn, E. J., Black, J. & Grayburn, P. A. Left ventricular hypertrophy associated with chronic cocaine abuse. *Circulation* **84**, 1130–1135 (1991).
10. Chakko, S. *et al.* Cardiac manifestations of cocaine abuse: a cross-sectional study of asymptomatic men with a history of long-term abuse of "crack" cocaine. *J. Am. Coll. Cardiol.* **20**, 1168–1174 (1992).

11. Roldan, C. A., Aliabadi, D. & Crawford, M. H. Prevalence of heart disease in asymptomatic chronic cocaine users. *Cardiology* **95**, 25–30 (2001).

12. Maceira, A. M. *et al.* Long term effects of cocaine on the heart assessed by cardiovascular magnetic resonance at 3T. *J. Cardiovasc. Magn. Reson.* **16**, 26 (2014).

13. Radunski, U. K. *et al.* Asymptomatic Cocaine Abuse—Myocardial Tissue Characterization Using Cardiac Biomarkers and Cardiovascular Magnetic Resonance Imaging—. *Circ. J.* **81**, 701–708 (2017).

14. Tong, W., Lima, J. A., Meng, Q., Flynn, E. & Lai, S. Long-term cocaine use is related to cardiac diastolic dysfunction in an African–American population in Baltimore, Maryland. *Int. J. Cardiol.* **97**, 25–28 (2004).

15. Carrillo, X. *et al.* Acute coronary syndrome and cocaine use: 8-year prevalence and inhospital outcomes. *Eur. Heart J.* **32**, 1244–1250 (2011).

16. Frustaci, A. *et al.* Oxidative myocardial damage in human cocaine-related cardiomyopathy. *Eur. J. Heart Fail.* **17**, 283–290 (2015).

17. Diercks, D. B. *et al.* Illicit stimulant use in a United States heart failure population presenting to the emergency department (from the Acute Decompensated Heart Failure National Registry Emergency Module). *Am. J. Cardiol.* **102**, 1216–1219 (2008).

18. Karch, S. B., Green, G. S. & Young, S. Myocardial hypertrophy and coronary artery disease in male cocaine users. *J. Forensic Sci.* **40**, 591–595 (1995).

19. Karch, S. B., Stephens, B. & Ho, C.-H. Relating cocaine blood concentrations to toxicity—an autopsy study of 99 cases. *J. Forensic Sci.* **43**, 41–45 (1998).

20. Mosunjac, M. I., Sundstrom, J. B., Heninger, M., Ansari, A. A. & Mosunjac, M. B. Combined pathological effects of cocaine abuse and HIV infection on the cardiovascular

system: an autopsy study of 187 cases from the Fulton County Medical Examiner's office. *Am. J. Forensic Med. Pathol.* **29**, 9–13 (2008).

21. Lucena, J. *et al.* Cocaine-related sudden death: a prospective investigation in south-west Spain. *Eur. Heart J.* **31**, 318–329 (2010).
22. Cigarroa, C. G., Boehrer, J. D., Brickner, M. E., Eichhorn, E. J. & Grayburn, P. A. Exaggerated pressor response to treadmill exercise in chronic cocaine abusers with left ventricular hypertrophy. *Circulation* **86**, 226–231 (1992).
23. Mondillo, S., D'Aprile, N., Nicoletti, A. & Palazzuoli, V. Valutazione del rilasciamento ventricolare sinistro nei pazienti dediti all'uso voluttuario di cocaina. *Minerva Cardioangiol.* **45**, 489–493 (1997).
24. Fineschi, V., Wetli, C. V., Di Paolo, M. & Baroldi, G. Myocardial necrosis and cocaine. *Int. J. Legal Med.* **110**, 193–198 (1997).
25. Fineschi, V. *et al.* Myocardial disarray: an architectural disorganization linked with adrenergic stress? *Int. J. Cardiol.* **99**, 277–282 (2005).
26. Nguyen, P. *et al.* Comparison of frequency of cardiovascular events and mortality in patients with heart failure using versus not using cocaine. *Am. J. Cardiol.* **119**, 2030–2034 (2017).
27. Egbuche, O. *et al.* Effect of β-blocker Therapy on Hospital Readmission and Mortality in Heart Failure Patients With Concurrent Cocaine Use. *J. Cardiovasc. Pharmacol. Ther.* 1074248418778550 (2018).
28. Lopez, P. D. *et al.* Clinical outcomes of B-blocker therapy in cocaine-associated heart failure. *Int. J. Cardiol.* **277**, 153–158 (2019).
29. Lopez, P. D. *et al.* Improvement in clinical outcomes of patients with heart failure and active cocaine use after β-blocker therapy. *Clin. Cardiol.* **41**, 465–469 (2018).

30. Kasper, E. K. *et al.* The causes of dilated cardiomyopathy: a clinicopathologic review of 673 consecutive patients. *J. Am. Coll. Cardiol.* **23**, 586–590 (1994).

31. Willett, D. L., Brickner, M. E., Cigarroa, C. G., Eichhorn, E. J. & Grayburn, P. A. Racial differences in the prevalence of left ventricular hypertrophy among chronic cocaine abusers. *Am. J. Cardiol.* **76**, 937–940 (1995).

32. Melon, P. G., Boyd, C. J., McVey, S. & Mangner, T. J. Effects of active chronic cocaine use on cardiac sympathetic neuronal function assessed by carbon-11-hydroxyephedrine. *J. Nucl. Med.* **38**, 451 (1997).

33. Mehta, S. K. *et al.* Diastolic filling abnormalities by color kinesis in newborns exposed to intrauterine cocaine. *J. Am. Soc. Echocardiogr.* **15**, 447–453 (2002).
